# Optimizing Scorpion Toxin Processing through Artificial Intelligence

**DOI:** 10.3390/toxins16100437

**Published:** 2024-10-11

**Authors:** Adam Psenicnik, Andres A. Ojanguren-Affilastro, Matthew R. Graham, Mohamed K. Hassan, Mohamed A. Abdel-Rahman, Prashant P. Sharma, Carlos E. Santibáñez-López

**Affiliations:** 1 Department of Biology, Western Connecticut State University, Danbury, CT 06810, USA; 2 División Aracnología, Museo Argentino de Ciencias Naturales, Buenos Aires C1405, Argentina; 3 Department of Biology, Eastern Connecticut State University, Willimantic, CT 06226, USA; 4 Zoology Department, Faculty of Science, Port Said University, Port Said 42521, Egypt; 5 Zoology Department, Faculty of Science, Suez Canal University, Ismailia 41522, Egypt; 6 Department of Integrative Biology, University of Wisconsin-Madison, Madison, WI 53706, USA

**Keywords:** python, RNAseq, sodium channel toxins, neural network

## Abstract

Scorpion toxins are relatively short cyclic peptides (<150 amino acids) that can disrupt the opening/closing mechanisms in cell ion channels. These peptides are widely studied for several reasons including their use in drug discovery. Although improvements in RNAseq have greatly expedited the discovery of new scorpion toxins, their annotation remains challenging, mainly due to their small size. Here, we present a new pipeline to annotate toxins from scorpion transcriptomes using a neural network approach. This pipeline implements basic neural networks to sort amino acid sequences to find those that are likely toxins and thereafter predict the type of toxin represented by the sequence. We anticipate that this pipeline will accelerate the classification of scorpion toxins in forthcoming scorpion genome sequencing projects and potentially serve a useful role in identifying targets for drug development.

## 1. Introduction

Scorpion venom is a complex mixture of inorganic and organic components such as polysaccharides, lipids, enzymes and short cyclic peptides (known as toxins) capable of modifying ion channels in target organisms [1,2]. The origin of these toxins has been traced to ancestral immune-related proteins such as defensins [3], (reviewed in [4]), and/or exon shuffling between ancestral venom gland genes and housekeeping genes [5]. All scorpions bear toxins, which are classified based on the ion channel they modify (e.g., sodium channel toxin, NaTx; potassium channel toxin, KTx), or by the type of fold by which they are stabilized (e.g., cysteine-stabilized α-helix and β-sheet fold: CSαβ; inhibitor cystine knot: ICK). The study and discovery of these toxins in scorpion venom has increased due to the availability of RNA sequencing for nearly 130 species in 17 scorpion families (i.e., [6,7,8,9]), in tandem with studies testing toxin expression using proteomics and mass spectrometric analyses (e.g., [10,11,12,13,14,15,16]). However, annotating toxins using in silico analysis remains challenging, mainly due to the number of peptides with validated functions as well as the short length of typical toxin sequences (e.g., [17,18,19]).

Annotating proteins in silico then becomes fundamentally a classification problem of assigning the sequence into a discrete class or assigning class labels to the protein. One of the main use cases for Artificial Intelligence (AI), such as a neural network approach, is for such classification problems [20]. As such, it is a natural fit to annotate transcriptomes with the aid of machine learning, assigning proteins to various classes for later analysis. Most transcriptomic analyses involve the Basic Local Alignment Search Tool (BLAST), analyzing sequences and annotating them by sequence similarity [21]. However, BLAST may not be able to distinguish paralogs from the desired proteins. A neural network approach can learn and understand what groups of residues mean, how they relate with each other, and what functions they might fulfill, allowing this approach to make more insightful and accurate predictions based on a higher-order data structure that can complement initial BLAST analyses (reviewed in [22]). As a result, using neural network approaches to classify, analyze, and annotate peptide and protein sequences is becoming a popular alternative to traditional methods such as BLAST. As examples, Wong et al. [23] successfully used support vector machines (SVMs) to predict propeptide cleavage sites in spider venoms. Similarly, Toxify [24] is a recurrent neural network using gated recurrent units that scores proteins based on the probability of being a toxin, being trained on the toxins of multiple taxa. Bileschi et al. [25] used deep learning models trained on sequences from the Pfam database to predict functional annotations for unknown sequences, extending the coverage of the Pfam database by >9.5%. Furthermore, AI has been used to successfully assign gene ontology terms to unannotated proteins [26]; Sanderson et al. [27] used convolutional neural networks (CNNs) to predict protein and enzymatic function of unknown sequences, with over 10,000 classification labels encapsulating these functions.

Here, we used a neural network approach to create a pipeline to validate the annotation of scorpion toxins (short cyclic peptides acting on ion channels) through methods like BLAST. The work carried out here uses comparatively small datasets and AI models than those previously mentioned, as well as newly sequenced scorpion venom transcriptomes. We aim to show that the generality and complexity of previous models can be replaced with simplicity and specificity, allowing AI models (like a neural network approach) to be custom-made for specific tasks with ease. The simplicity of these models herein also reduces computational and time requirements, removing barriers for building and training AI models.

## 2. Results

### 2.1. Tapai (Transcriptome Processing by Artificial Intelligence), a Python Neural-Network-Approach Script to Classify Scorpion Toxins

**Figure 1:**
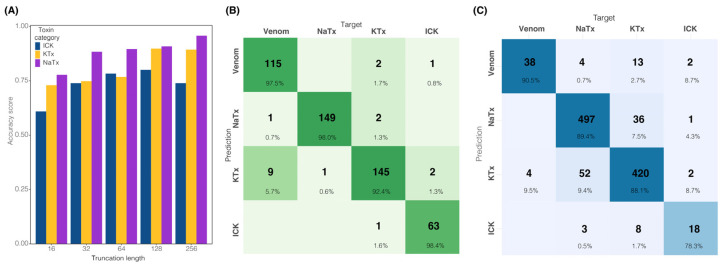
Length cut-off effect on training data and *tapai* performance. (**A**) Validation accuracy for different peptide truncation/padding lengths. Results of sequence truncation length on the validation accuracy of the toxin model. (**B**) Confusion matrix showing *tapai* performance with the complete dataset (validation and testing sets). (**C**) Confusion matrix showing *tapai* performance with sequence truncation length to 128 residues. Color intensity in (**B**,**C**) represents the percentage of correct classifications for each combination of predicted and actual classes. Four additional toxin models were created with the TV layer truncating or padding to 16, 32, 64, and 256 residues, and trained using the same hyperparameters (Figure S1).

Here, we present *tapai* (https://github.com/Adampse/Tapai, accessed on 26 June 2024), a neural-network-approach python script for scorpion toxin classification using amino acid sequences in fasta format. To facilitate the training of neural-network-approach models for the pipeline, a new dataset was created retrieving all scorpion venom peptides from the UniProt database and recently published scorpion venom datasets (File S1). Peptides were classified into four categories: toxins with the inhibitor cystine knots (ICKs), potassium (KTx) and sodium (NaTx) channel toxins (known afterward as scorpion toxins), and venom proteins (e.g., all those components that do not act on ion channels like venom allergens). A fifth category (housekeeping genes) was created to include all those peptides that have a non-enzymatic effect on target organisms (e.g., the *Drosophila melanogaster* transcriptome was used as a negative contrast against the toxin dataset). The training set of the Toxify dataset [24] was also used for validation testing, although modified to remove sequences that were already present in the *Drosophila* transcriptome and the toxin dataset. This left 5453 sequences from the original 6133 in the positive Toxify dataset, and 49,694 sequences in the negative Toxify set out of the original 50,000. The training model required a fixed-sized input; we therefore tested five toxin models that were made by truncating or padding sequences to 16, 32, 64, 128, and 256 residues. Confusion matrices and the performance of all truncation analyses are shown in Figure S1. Selecting 128 residues as the basis for model evaluation, the housekeeping model was able to achieve 99.46% validation accuracy for the toxin dataset and 94.44% for the *Drosophila* dataset, as well as 74.55% on the modified positive Toxify set and 89.92% validation accuracy on the modified negative Toxify set. The toxin model had 80%, 85.11%, 93.88%, and 92.86% validation accuracy for the ICK, KTx and NaTx, and venom peptide classes, respectively (Figure 1). The training and validation datasets had a Matthews Correlation Coefficient (MCC) of 0.947 and 0.798, respectively. These percentages and MCCs correlate with the number of sequences used in their training datasets, as shown in Table 1.

### 2.2. RNA Sequencing and Transcriptome Assembly, and Toxin Classification

To test our pipeline, we retrieved SRA datasets from the NCBI database and assembled 10 published transcriptomes (using Trinity v.2.5 [28]) along with sequencing new scorpion venom transcriptomes from two bothriurid species (*Brachistosternus diaguita* and *Urophonius tregualemuensis*) and one anuroctonid (*Anuroctonus phaiodactylus*; Table 2).

Initial de novo annotation of toxin transcripts for the three species sequences was performed with BLASTp, and a database comprising selected toxin sequences (File S2) recovered 4 (*A. phaiodactylus*), 14 (*B. diaguita*), and 63 transcripts (*U. tregualemuensis*) with percentages of similarity between 50 and 100% and e-values ranging from 1 × 10^−10^ to 1 × 10^−60^ (Figure S2). Using the same procedure as above, we retrieved 741 transcripts from the 10 published transcriptomes (Table S1).

These sequences were then processed one species at a time using our new program *tapai* which classified them as follows: 29 transcripts as ICK, 335 as KTx, and 464 as NaTx. Lastly, 49 transcripts were classified as venom peptides (venom). The species with most toxin transcripts annotated were *Androctonus mauritanicus* (with 189 transcripts), followed by *Centruroides sculpturatus* (with 173 transcripts) and *Centruroides limpidus* (with 100 transcripts; Table S1), whereas the species with the least transcripts annotated were *A. phaiodactylus* (with four transcripts), *Centruroides vittatus* (with 10 transcripts), and *B. diaguita* (with 14 transcripts).

To explore whether the size of transcripts (~80 to 3000 bp) influences toxin classification in *tapai*, we removed sequences with more than 200 bp using a python script from the database for each species, keeping only 817 sequences for another round of search using *tapai*. From these transcripts, 16 were classified as ICK, 244 as KTx, 488 as NaTx, and 23 as hypothetical venom peptides (Table S1, File S3). To assess for statistical differences in removing (or not) transcripts longer than 200 amino acids, we conducted t-tests on the means of transcripts annotated by these two analyses (reduction before *tapai* vs. no reduction). These tests suggest that there is no significant difference between removing the sequences longer than 200 amino acids before running *tapai* (Figure S3).

**Figure 2:**
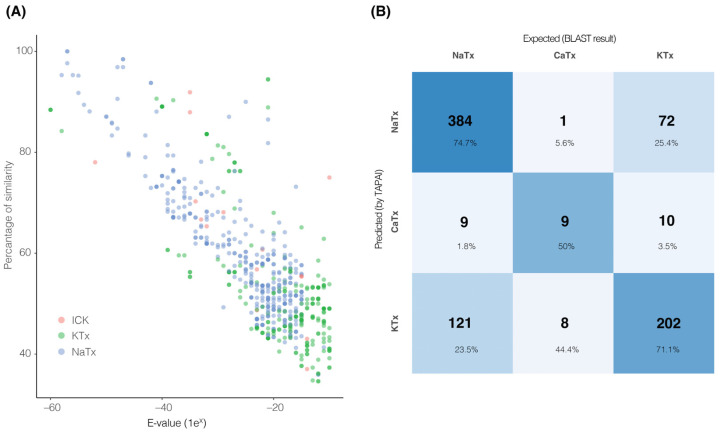
BLAST sequence similarity and *tapai* performance. (**A**) The distribution of percentage of similarity and e-values from the initial BLAST analysis plotted as a function of type of toxin (ICK: red, KTx: green, NaTx: blue). (**B**) Confusion matrix showing the classification performance of *tapai* in comparison to BLAST similarity predictions.

**Figure 3:**
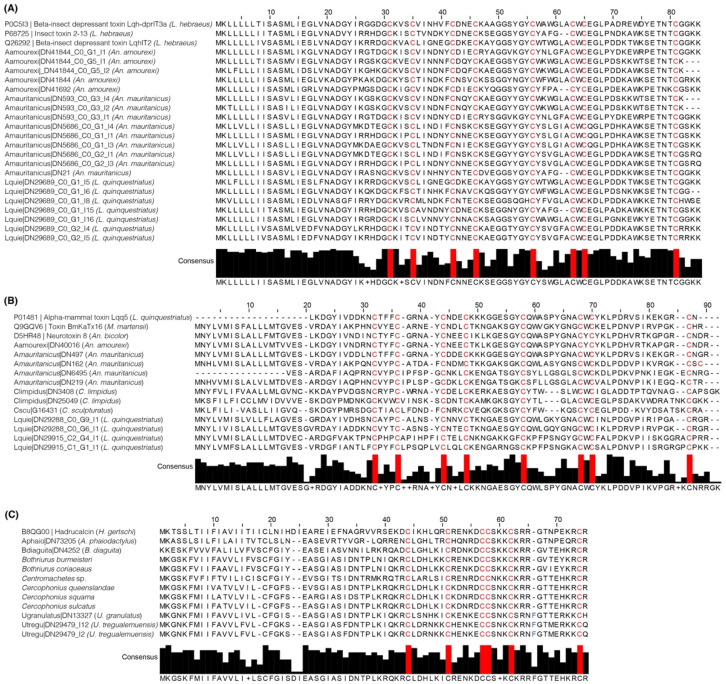
Multiple sequence alignment (MSA) of transcripts classified by *tapai* as (**A**) NaTx with “only insect” affinity, (**B**) NaTx with “only mammal” affinity (all from buthid scorpions), and (**C**) calcins (all from iurid scorpions). Top sequences (those with accession numbers) on each MSA were retrieved from the UniProt. Consensus sequence histograms are found below each MSA (red color indicates the conservative cysteine pattern).

Next, we compared the number of transcripts recovered by *tapai* against the number of transcripts used as input (those recovered from the BLAST analysis) to assess the differences between the two pipelines (annotation using BLAST only vs. using *tapai*). First, we explored the distribution of percentage of similarities between the sequences and the queries, and the e-values recovered from the initial search (Figure 2A and Figure S2). As shown in Figure 2A, the distribution of high e-values and low percentages of similarity in KTx peptides suggests that these peptides are hard to identify through similarity only. By contrast, ICK had higher percentages of similarity and lower e-values. Second, the same number of transcripts recovered through BLAST in 2 out of 13 species were recovered by *tapai*. In 11 species, *tapai* removed the following number of transcripts: (a) one in *C. hirsutipalpus* and *C. limpidus*, two in *B. diaguita*, *C. hentzi* and *C. vittatus*, three in *An. amourexi* and *U. tregualemuensis*, five in *C. sculpturatus*, thirteen in *An. mauritanicus*, twenty in *T. serrulatus*, and twenty-six transcripts in *U. yaschenkoi*. Further, we compared the annotation results per category per species between BLAST and *tapai* (i.e., change in the classification of a transcript, visualized as a confusion matrix with the predicted result suggested by *tapai* and the expected result suggested by BLAST in Figure 2B). The most common change was changing the similarity of a transcript by BLAST as a NaTx to KTx by *tapai* (121 transcripts, Table S2), with only one transcript changed from ICK to NaTx (in *C. hentzi*). Per species, *T. serrulatus* was the species that had the most changes (21 transcripts out of 28; 75%), with the most common change being from KTx (determined by BLAST) to NaTx (determined by *tapai*; Table S3). On the other hand, *A. mauritanicus* and *C. hentzi* were the species with the least changes (5% of their transcripts; Table S3). Further scorpion NaTx classification used the transcripts recovered per species from the first analysis with *tapai* as input. These transcripts were processed with a modified *tapai* training dataset to classify them based on the function. The specific functions were (a) affecting only insect sodium channels, (b) affecting only mammal sodium channels, or (c) affecting both insect and mammal sodium channels. In total, from the 10 species, we recovered 34 sequences putatively affecting insect sodium channels only (from 6 species, Figure 3A), 62 putative sequences acting only on mammalian sodium channels (from 12 species, Figure 3B), and 120 putative sequences acting on both insects and mammals (Table S4, File S3).

To corroborate the identity of these NaTx sequences and assess the error rate in our pipeline, we performed BLAST searches on these sequences against the NCBI database. Of the 216 transcripts recovered in the previous step, 25 had a different identity from NaTx (11.57%), with 4 of these transcripts identified as having similarity to calcium channel toxins (U8-Agatoxin like peptides), 10 transcripts having similarity to diverse types of KTx peptides, and 11 transcripts having similarity to a diverse type of venom peptides. Interestingly, nine of the ten peptides with identity to KTx were recovered as matching KTx peptides in our first BLAST analysis. Analysis per species showed that the species with most mismatches was *U. tregualemuensis* with six mismatches out of thirteen transcripts (two in the category of toxins acting on both “insect and mammal” ion channels, and four in the “mammal only” category). *Androctonus mauritanicus* had four mismatches out of thirty transcripts (two in the “both insect and mammal” category, and two in the “only mammal” category). *Centruroides limpidus* had four errors out of thirty-two, all of them in the “only mammal” category. Lastly, the two species with the least number of mismatches were *C. hentzi* (one out of eleven in the “only mammal” category) and *L. quinquestriatus* (one out of twenty-six in the “only mammal” category).

### 2.3. Toxin Annotation on the Three New Scorpion Venom Transcriptomes

Using this pipeline, we report here 4 transcripts with toxin identity found in the venom gland transcriptome of *A. phaiodactylus*, 16 transcripts with toxin identity found in *B. diaguita*, and 64 transcripts with toxin identity found in *U. tregualemuensis*. From the six transcripts found in *A. phaiodactylus*, *tapai* classified transcript Aphaio|DN5787 within the ICK class. However, BLAST analysis showed this transcript had sequence similarity to Phi-liotoxin-Lw1a (UniProt accession number P0DJ08). Transcripts Aphaio|DN3807_i2 and Aphaio|DN3807_i3 were classified by *tapai* as members of the KTx class, confirming the BLAST result (both sequences had similarity to Hge-scorpine (UniProt accession number Q0GY40). A third transcript (Aphaio|DN39572) was classified as member of KTx, but BLAST analysis showed this transcript had 48% of similarity to Phi-liotoxin-Lw1a. From the fourteen transcripts found in *B. diaguita*, two were classified within the ICK class, Bdiaguita|DN598_i5 and Bdiaguita|DN598_i9, both showing sequence similarity with Hge-scorpine (two mismatches between *tapai* and BLAST results as reported above, Table S5). Five more transcripts found in *B. diaguita* were classified within the KTx class by *tapai*: (a) Bdiaguita|DN598_i1, B. diaguita|DN513_i3 and B. diaguita|DN513_i4 were recovered similarly to the Hge-scorpine by BLAST; (b) Bdiaguita|DN1686 had sequence similarity to a kappa KTx (UniProt accession number P0DJ41); and (c) Bdiaguita|DN85245 was recovered with similarity to a putative Agatoxin-like toxin (UniProt accession number A0A224X3X6), representing another ICK to KTx mismatch. The last seven transcripts were classified as members of the NaTx class by *tapai*: BLAST showed that transcripts (a) Bdiaguita|DN3450 had similarity to the Alpha-insect LqhaIT (UniProt accession number P17728) (b) Bdiaguita|DN34333 and Bdiaguita|DN39645 had similarity to the Alpha-mammal-toxin-Lqq5 (UniProt accession number P01481). Further, there were four mismatches: transcripts Bdiaguita|DN598_i2, Bdiaguita|DN598_i3, Bdiaguita|DN513_i4 and Bdiaguita|DN513_i11 were classified as members of this class (NaTx) but they had similarity to Hge-scorpine by BLAST (mismatch KTx to NaTx).

Lastly, from the 63 transcripts recovered from the *U. tregualemuensis* transcriptome, 5 were classified as ICK, 32 as KTx, and 26 as NaTx. From the five transcripts classified as ICK, BLAST recovered similarities to these transcripts (Utregu|DN29479_i2, Utregu|DN29479_i10, Utregu|DN29479_i12 and Utregu|DN29479_i22) to Hadrucalcin (UniProt accession number B8QG00), and one mismatch (Utregu|DN94692, from KTx to ICK). Further, from the thirty-two putative KTx transcripts, BLAST recovered similarities of fourteen transcripts to the Kunitz-type serine protease LmKTT-1a (UniProt accession number P0DJ46)*;* seven transcripts to gamma KTx 1.1 (UniProt accession number Q86QT3); five transcripts to kappa KTx (P0DJ41); and six transcripts to Hge-scorpine (Q0GY40). Lastly, from the twenty-six transcripts classified as NaTx by *tapai*, BLAST found similarities of nineteen transcripts to the Alpha-mammal toxin Lqq5 (UniProt accession number P01481); one transcript to the Alpha-insect toxin Lqq3 (UniProt accession number P0148); and six transcripts with a mismatch (KTx to NaTx, four of them with similarity to the Kunitz-type serine protease LmKTT-1a, and two of them with similarity to Hge-scorpine).

Since calcins are phylogenetically restricted to the clade Iurida (e.g., [29]), it is interesting that these peptides were not found in the venom transcriptomes of *A. phaiodactylus* and *B. diaguita* (BLAST initially did not obtain significant hits with lower e-values than 1 × 10^−1^). To rule out the lack of expression of these peptides in the transcriptome of these species, and to assess the impact of the translation process (through TransDecoder [30,31]) in translating small peptides like calcins, we searched for these peptides in the nucleotide assemblies using tBLASTn and the Hadrucalcin peptide as a query (B8QG00). We recovered one transcript from *A. phaiodactylus* with 58% similarity to Hadrucalcin and one transcript from *B. diaguita* with 66% similarity to Hadrucalcin. These peptides are compared to other putative calcins in Figure 3C.

## 3. Discussion

In this contribution, we introduced a new pipeline to annotate scorpion transcriptomes using a neural network approach (available at https://github.com/Adampse/Tapai). This approach uses two models (housekeeping and channel models) which are small convolutional networks that pad or truncate input sequences to a length of 128 amino acid sites, with each being under 31,000 parameters. The training model required a fixed-sized input; we tested four extra toxin models that were made by truncating or padding sequences to 16, 32, 64, and 256 residues. At a threshold of 256 residues, the model did not yield any substantially greater validation accuracy than 128 residues (78–91% with 128; 74–95% with 256; Figure 1B and Figure S2). Further, a significant drop in performance was observed at 32 and 16 residues compared to 128 residues (69–77% with 16, 74–88% with 32, and 77–90% with 64). The use of 64 residues yielded minor drops in performance compared to 128, although with slightly better validation accuracy on calcium sequences (Figure 1B and Figure S2). The variable length of sequences does not appear to hinder models that require fixed inputs. It was also found that the validation split of the toxin dataset could drastically affect validation accuracy for each class (Table 3). The sodium channel toxin class was the most stable in terms of validation accuracy, suggesting that many sodium channel toxins are similar. The ICK class showed larger variance, which likely partly reflects the more limited training data (less than half the available sequences compared to the sodium channel class). However, the potassium channel toxins, despite having 150 training sequences (just as with sodium channel toxins), also showed greater deviation in validation accuracy dependent on the validation split. This suggests that the category “potassium channel toxins” is more variable in sequence composition than the sodium channel toxins, as suggested by several subclassifications including potassium channel toxins (KTxs), scorpines, and Kunitz-type inhibitors (see [8]). The venom class also showed large deviations in validation accuracy, suggesting that these sequences are also quite disparate, although the comparative lack of validation sequences makes the comparison harder. Lastly, our program also allows users to train new models on data for specific tasks.

We did not perform a redundancy reduction of the sequences, as our model here is already small and computationally fast. Based on Geron (2017), if we reduce the dimensionality of our training set before training the model, it will speed up this process; however, it may not lead to a better solution. Similarly, the small dataset used here (i.e., low number of ICK sequences) prevents running a separate test set. As a workaround, we performed hyperparameter model tuning of the training set.

The mismatches between the results from BLAST and *tapai* in our only NaTx analysis could be attributed to the presence of under-specification in some of the training sequences. The ensemble methodology with the insect/mammal models was carried out as each individual model had vastly different predictions on unknown sequences. Under-specification is the result of a neural network learning spurious associations present in the training when validation data do not exist outside of this dataset. This is a common issue and the largest reason why many machine learning and neural-network-approach models have subpar performance when initially deployed [32]. Due to the low number of training sequences, it was likely that the neural network approach could not learn enough and was biased by the sequences it was trained on; the models could not grasp a fuller or more meaningful understanding of the data. Thus, using an ensemble of the three models engenders greater confidence in the predictions of the final sequences. However, this is a suboptimal solution due to the discarded sequences and improvements to the method (e.g., pseudoaugmentation) should be examined in future iterations.

## 4. Conclusions

Our study employed basic neural networks to analyze scorpion venom transcriptomes. This approach facilitated the identification and classification of putative toxin sequences, along with predictions regarding their toxin type. Notably, our AI implementation significantly expedited the classification of toxins based on sequence similarity. More importantly, by leveraging a “function-based” classification model, we open a venue for enhanced studies in peptide synthesis. This approach enables the testing of specific venom components with promising applications in drug development and the creation of more efficient antivenoms. The implementation of *tapai* is anticipated to fulfill a downstream role to approaches like Toxify, with the latter classifying toxin sequences out of a large transcriptomic input and *tapai* further classifying the toxins into functional categories.

## 5. Materials and Methods

### 5.1. Biological Material, RNA Extraction, RNA Sequencing and Transcriptome Assembly

Scorpion specimens were collected in several localities in the United States, Chile, and Argentina (see Table S6). Total RNA was extracted using the RNA extraction kit (QIAGEN) from the venom glands of one adult female of the species *Anuroctonus phaiodactylus*, one adult male of the species *Brachistosternus diaguita*, and one adult male of the species *Urophonius tregualemuensis* following the manufacturer’s protocol. RNA library construction and paired-end transcriptome sequencing on the Illumina HiSeq 2500 platform were performed for these three species at the UW-Madison Biotechnology Center (Madision, WI, USA). Newly generated transcriptomes were assembled using Trinity v.2.5 [28], removing the adaptors with Trimmomatic v.0.36 [33] and assessing the quality of cleaned raw reads with FastQC v.0.11.5 [34]. Protein-coding regions within the assembled transcripts were identified using TransDecoder v.5.3.0 [30,31]. Toxin annotation was conducted with the NCBI BLAST suite as follows: a database was created for each of the three newly generated transcriptomes plus 10 published transcriptomes (Tables S7 and S8), using a query comprising well-studied toxins (File S2).

### 5.2. AI Processing Pipeline

To facilitate the training of our neural-network-approach models, for the pipeline, a new dataset was created. Reviewed sequences from scorpions were gathered from Uniprot’s Toxin annotation project along with published transcriptomes, and sorted into four classes: sodium, potassium, ICK, and a generic venom class for miscellaneous sequences. Chloride toxins were grouped together with potassium toxins due to their close evolutionary distance [8]. This set was designated as the “toxin dataset” (Table 1). A second dataset of toxins affecting insects, mammals, or both was assembled and used to train AI to classify toxins into one of the three classes. This dataset was designated as the “insect and mammal” dataset (Table 2). The AI model trained on the toxin dataset was termed the toxin model and the AI models trained on the insect–mammal dataset as the insect–mammal models. All models used are basic CNNs using one-dimensional convolution layers, with the toxin model being 30,772 parameters and the insect–mammal models being 31,587 parameters (File S4).

The toxin model was trained on the toxin dataset to sort into the four classes of sodium, potassium, ICK, and other venom peptides. Due to the limited data and uneven class distribution, class-wise stratification was used. Stratification was performed by first applying the validation split of 0.25 to each class. Then, if the training set for that class was over 150 sequences (150 + X number of sequences), X sequences were taken from the training set and appended to the validation set to cap the training set to 150 instances. The number of training instances for the insect–mammal dataset for each class was set to 32 sequences with no previous validation split.

These sequences were converted from fasta files to CSV files (available at our GitHub repository), with two columns of class and sequence. Each residue symbol in the sequence is separated by whitespace for input into an AI model using Keras’s TextVectorization layer (TV layer). A custom dropout layer, IntDropout (see File S4), was used in the toxin model to mask residues prior to embedding. Due to the low number of sequences for “Only mammal” active classes and “Only insect” active classes, 42 sequences each (Table 2), three identical neural-network-approach models were built and trained using different validation sets. These three models were then used to give a consensus when used on transcriptomes. Each model had to predict a sequence to the same class otherwise it would be discarded.

All models were built using Keras 2.11 [35] and TensorFlow 2.11 [36] and ran using scripts made with Python 3.8.12, Pandas 1.2.4 [37,38], and NumPy 1.20.2 [39]. All models were built using Keras and compiled with the sparse categorical cross-entropy loss function, accuracy as the validation metric, and an Adam optimizer with a learning rate of 0.001. In all models, sequences were either truncated or padded to a length of 128 residues using the TV layer, and the final activation was SoftMax. The dropout rate for both the toxin model and the insect–mammal models was set to 0.35 for all dropout layers including the custom IntDropout layer. Training of the toxin model was carried out for 24 epochs with a batch size of 32. Training of the insect–mammal models occurred for eight epochs with a batch size of eight. All training occurred on an AMD R5 2600 CPU with 16 GB of RAM.

After training, the models were tested on the selected transcripts recovered from the BLAST annotation from 13 transcriptomes (as mentioned above).

## Figures and Tables

**Table 1:** Per-class breakdown of the toxin dataset with the number of training and validation sequences per class used to train the toxin model.

Class	Total	Training Sequences	Validation Sequences
Calcins|DDH (ICKs)	89	66	23
Potassium channel toxins (KTxs)	627	150	477
Sodium channel toxins (NaTxs)	706	150	556
Other venom proteins (venom)	167	125	42

**Table 2:** Per-class breakdown of the NaTX insect/mammal dataset with the number of training and validation sequences per class used to train the “insect and mammal toxins” models.

Class	Total	Training Sequences	Validation Sequences
Toxin acting on both insect and mammal sodium channels	908	32	876
Toxins acting only on insect sodium channels	42	32	10
Toxins acting only on mammal sodium channels	42	32	10

**Table 3:** Validation accuracy per class with the toxin model trained over eight random validation splits along with mean and standard deviation (SD). Values provided are percentages.

Class	Split 1	Split 2	Split 3	Split 4	Split 5	Split 6	Split 7	Split 8	Mean	SD
Calcin|DDH (ICK)	100	82.61	86.95	78.26	82.61	91.30	82.61	82.60	85.87	6.87
Potassium (KTx)	82.18	89.52	73.79	77.78	87.63	78.41	76.73	71.07	79.64	6.43
Sodium (NaTx)	96.94	91.19	96.94	95.86	88.13	92.99	92.99	97.66	93.82	3.53
Venom	69.05	78.57	64.29	83.33	71.43	71.43	71.43	73.81	74.70	7.30

## Data Availability

This pipeline is available at https://github.com/Adampse/Tapai. Further details about the script are found in our repository.

## Supplementary Materials

The following supporting information can be downloaded at https://www.mdpi.com/article/10.3390/toxins16100437/s1.
