# Supplementary material for: Optimizing Scorpion Toxin Processing through Artificial Intelligence
Source: Toxins (Basel). 2024 Oct 11;16(10):437. doi: 10.3390/toxins16100437 (PMC11511117; doi:10.3390/toxins16100437)
Supplement: Supplementary file 1 [file toxins-16-00437-s001.zip › Supplementary_data/Supplementary_Figures.pdf]

Supplementary Figures

Optimizing scorpion toxin processing through artificial intelligence

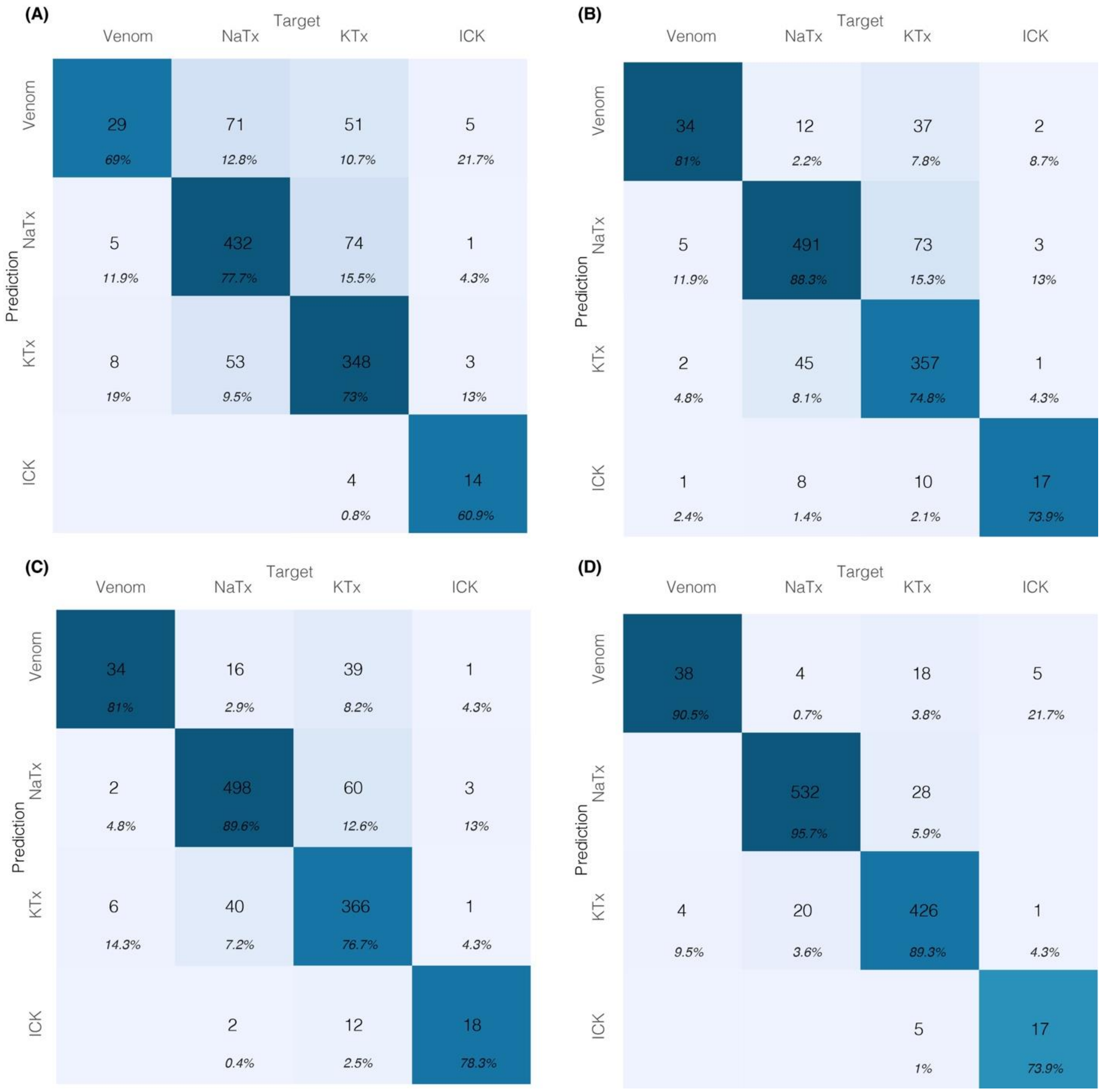

Figure S1. tapai learning model performance comparison. Confusion matrices showing *tapai* performance at different sequence truncation length on the validation accuracy of the toxin model (categories ICK, NaTx, KTx, and Venom only): (A) 16, (B) 32, (C) 64, and (D) 256.

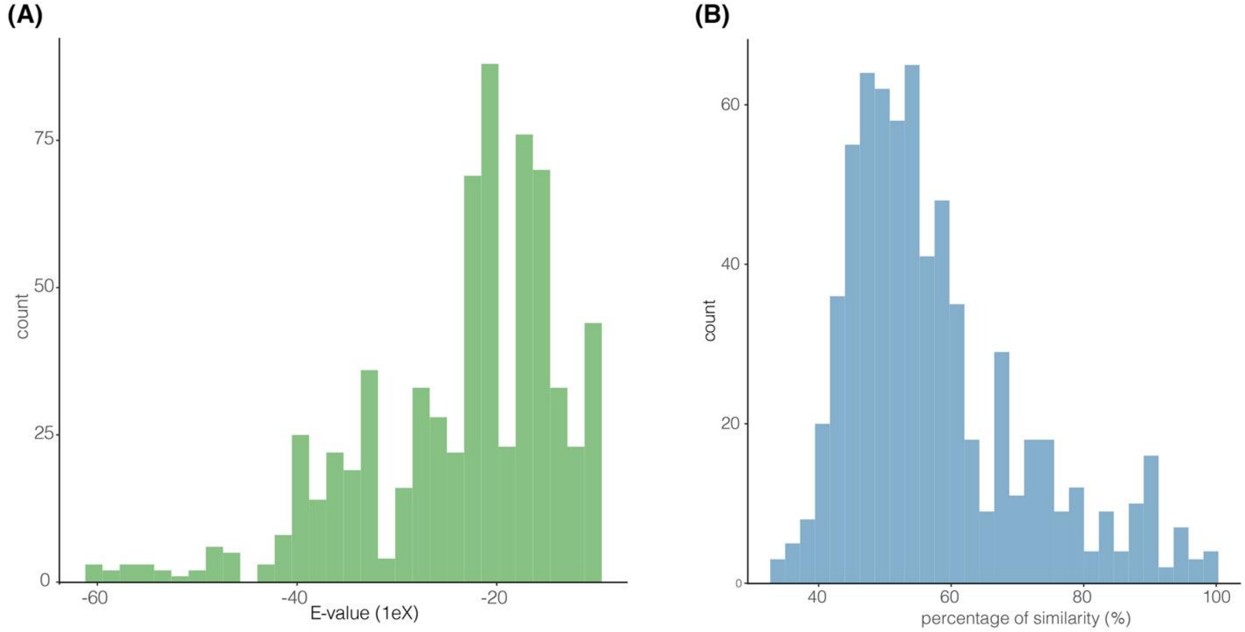

Figure S2. Sequence similarity and e-values distribution as recovered by initial BLAST analyses. (A) Distribution of the e-values retrieved from the initial blast toxin annotation. (B) Distribution of sequence similarity retrieved from the initial blast analysis.

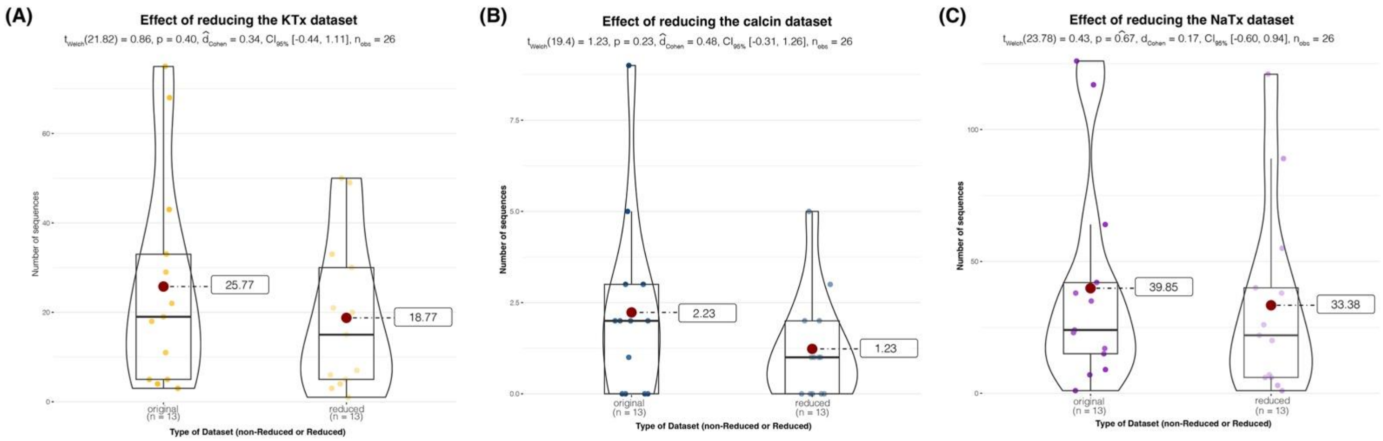

Figure S3. Assessing the differences between the mean of the number of putative calcin/DDH (ICK, A), potassium channel toxins (KTx, B), and sodium channel toxins (NaTx, C) sequences retrieved from *tapai* analyses without removing transcripts with more than 200 amino acids ("original"), and the *tapai* analyses with transcripts with more than 200 amino acids removed a priori ("reduced").
